# Bird flu in the spotlight: A Thanksgiving special issue

**DOI:** 10.1093/immhor/vlaf059

**Published:** 2025-11-24

**Authors:** Bonnie N Dittel

**Affiliations:** Editor-in-Chief, ImmunoHorizons

As I sit on my back deck enjoying the crisp fall air, admiring my garden where wild things grow, I can’t help but think of the neighborhood turkeys I spotted down the road this past summer. Will they peck at my flowers, scratch up my lawn, or strut through like they own the place? Whatever they bring, it’s likely more chaos, joining the ranks of deer, chipmunks, and rabbits. But beyond their beaks and claws, these turkeys may harbor something far more concerning: avian influenza.

H5N1 influenza virus is no joke. It infects turkeys and continues to cause a panzootic, devastating poultry farms and leading to the culling of thousands of birds. In honor of Thanksgiving and the turkeys that make it to our tables, we present the inaugural *ImmunoHorizons* themed collection, timed perfectly with the season. This special issue features 3 comprehensive reviews from leading investigators with an emphasis on bird flu.

*Peña-Hernandez and colleagues* ^1^ examine the potential for H5N1 to expand into humans, a critical concern as infected birds have now been detected across the United States. While human infections remain rare, the expansion of host populations to farm animals, such as cows and pigs, underscores the emerging zoonotic threat.

*Chou and colleagues* ^2^ focus on highly pathogenic avian influenza and explore how ecosurveillance combined with predictive modeling could anticipate and mitigate spillover events. Their review provides a clear framework for preventing the next potential pandemic.

*Chen and colleagues* ^3^ describe the innovative use of human immune organoid cultures to study T memory responses to influenza, leveraging high-dimensional immune profiling to find new vaccine candidates. This cutting-edge approach exemplifies how functional systems immunology can help us prepare for future influenza outbreaks.

Of course, no editorial about turkeys would be complete without a nod to the birds on our tables. I love turkey, and after years of trial and error, I’ve perfected my method: brining with herbs, stuffing with an apple and herb family recipe, cooking it the day before, and letting it rest well before carving. On Thanksgiving, I gently rewarm the turkey and stuffing with a splash of chicken broth. The result? Moist, flavorful turkey and a stress-free holiday, ready to be enjoyed with family and friends.

As you explore this special bird flu collection, I hope you gain insight into the mechanisms, risks, and innovative strategies to combat this virus. I also wish that your Thanksgiving is full of good company, good food, and perhaps a turkey-free encounter with H5N1.
